# Qualitative Analysis of Constructional Errors in Neurodegenerative Conditions: A Systematic Review

**DOI:** 10.3390/brainsci16070667

**Published:** 2026-06-25

**Authors:** Vincenzo Crisci, Laura Sagliano, Antonella Ferrara, Alessia Salzillo, Luigi Trojano, Francesco Panico

**Affiliations:** Department of Psychology, University of Campania “Luigi Vanvitelli”, Viale Ellittico 31, 81100 Caserta, Italy; vincenzo.crisci@unicampania.it (V.C.); laura.sagliano@unicampania.it (L.S.); antonella.ferrara1@unicampania.it (A.F.); alessia.salzillo1@unicampania.it (A.S.); francesco.panico@unicampania.it (F.P.)

**Keywords:** constructional apraxia, dementia, mild cognitive impairment, neurodegenerative disorders, qualitative approach

## Abstract

**Highlights:**

**What are the main findings?**

- Different dementia syndromes show partially distinct qualitative constructional error profiles involving visuospatial, executive, attentional, and semantic processes.
- Spatial errors and simplifications are the most frequent constructional deficits reported across neurodegenerative disorders.

**What are the implications of the main findings?**

- Qualitative analysis of drawing and copying errors may support differential diagnosis when integrated with the broader neuropsychological profile.
- Standardized classification systems for constructional errors are needed to improve comparability across studies and clinical utility.

**Abstract:**

**Background/Objectives**: Constructional apraxia (CA) is an impairment in combining simple elements into coherent spatial configurations without basic motor deficits. Although common in neurodegenerative disorders, the qualitative features of visuo-constructional errors and their role in differentiating dementia types remain unclear. This systematic review aimed to synthesize patterns of visuo-constructional errors in dementia and mild cognitive impairment (MCI), exploring distinctive qualitative features associated with different neurodegenerative conditions. **Methods**: A systematic literature search was conducted in PubMed, Scopus, and Web of Science for studies published between January 1990 and January 2026, following PRISMA guidelines. Studies on adults with dementia or MCI assessing drawing/copying abilities through standardized tasks and qualitative error analysis were included. Reviews, meta-analyses, case reports, non-English articles, and studies not explicitly assessing constructional apraxia were excluded. The quality of evidence was assessed using an adapted version of the Newcastle–Ottawa Scale. **Results**: A total of 25 studies were included, showing heterogeneous and condition-specific visuo-constructional deficits. Spatial errors and simplifications were the most common across disorders, while perseverations, rotations, and closing-in phenomena were less frequent. Alzheimer’s disease was mainly associated with spatial disorganization, omissions, and conceptual errors linked to temporo-parietal dysfunction; frontotemporal dementia with executive deficits such as perseverations and planning impairments; Lewy body and Parkinson’s disease dementias with visuospatial and attentional alterations; and Huntington’s disease with simplifications and executive dysfunction related to fronto-striatal involvement. **Conclusions**: No single error pattern was pathognomonic, but qualitative assessment of constructional errors may provide clinically useful information when integrated with the broader neuropsychological profile.

## 1. Introduction

Visuo-constructional abilities refer to the ability of integrating visual and spatial information through the planning, production and monitoring of the execution of bidimensional and tridimensional structures, when drawing spontaneously, copying figures or assembling objects [1]. An impairment of these abilities, termed constructional apraxia (CA), is defined as a difficulty in combining simple elements into coherent spatial configurations, in the absence of basic motor deficits [2]. Liepmann [3] had previously proposed that the execution of complex actions is contingent on organized motor representations, while Kleist [4] was the first to propose a formal distinction between CA and other forms of apraxia, such as limb apraxia.

Drawing spontaneously and copying have been the most used methods to assess visuo-constructional abilities since early studies [5]. Typical copying tasks require the reproduction of cubes and other geometric figures (e.g., the Constructional Apraxia Test, CAT [6]; the Beery–Buktenica Developmental Test of Visual–Motor Integration, VMI, ref. [7]), of lines with embedded elements [8,9], of two interlaced pentagons (from the Mini-Mental State Examination, ref. [10]), or of more complex stimuli such as the Rey–Osterrieth Complex Figure (ROCF, ref. [11]), the Taylor Figure [12], and the complex figure from the Birmingham Cognitive Screen (BCoS, ref. [13]). Visuo-constructional skills can also be assessed by drawing from memory, and in this case the most used tool is the Clock Drawing Test (CDT, ref. [14]). Other tasks require assembling three-dimensional structures but have received limited attention in clinical practice [15,16].

Over time, interpretations of CA have emphasized the role of the right [17] or the left hemisphere [18], as well as of the frontal and parietal lobes subserving executive and visuo-spatial functions [19,20]. Indeed, visuo-constructional abilities do not constitute a single domain, but depend on the interaction of multiple processes, including visual and perceptual processing, spatial organization, attention, planning and motor control [21]. For instance, visuo-perceptual processing enables the analysis of stimulus’ characteristics, visuo-spatial processes allow the encoding of relationships between parts, executive functions support planning and monitoring, attention enables the selection of relevant information, and the motor systems are involved in graphic execution [5]. As revealed by functional neuroimaging studies, these processes largely rely on a distributed and bilateral fronto-parietal network with connections to subcortical regions [22,23]. Within this network, the parietal lobes play a central role in visuospatial integration [24], while frontal areas contribute to planning and control [25].

Reduced accuracy in visuo-constructional tasks may be associated with distinct qualitative error patterns. Kleist [4] underlined that CA must be characterized by ‘spatial’ errors, but a more systematic description of constructional errors was later provided by Critchley [2] and can be schematized as follows, including some kinds of errors not considered at the time: (i) untidy execution, not properly related to constructional apraxia but rather to other motor disturbances (distorted/unsteady lines, size distortion—most often size reduction, lines intersecting each other or failures in joining lines); (ii) spatial misplacement/poor centering (i.e., crowding figures in one corner of the page), which together with lateralized omission of details can be suggestive of neglect-related disturbances; (iii) tendency to overlap with or to approach the model, termed closing-in after Mayer-Gross [26]; (iv) spatial-related errors, including errors in line orientation (i.e., making vertical lines oblique, or tilting horizontal lines), and abnormal elongation of the horizontal or vertical dimension; (v) mirror reversal of figures or their parts (which might be considered an instance of rotation errors); (vi) graphic perseverations, i.e., inappropriate repetition of elements; (vii) loss of perspective in drawing solid figures in the bidimensional plane, which might also include production of simplified figures instead of more complex items, or figures lacking some stimulus’ details (simplifications).

This kind of classification has not been adopted systematically, and the description of constructional errors varies greatly among authors, and also because the interpretation of constructional errors is not a straightforward procedure [27]. Nonetheless, the basic seven categories (untidy execution, neglect-related errors, spatial-related errors, simplifications, rotations, perseverations, closing-in) borrowed from Critchley [2] can be useful for analysis of constructional errors and of their neural correlates in copying tasks. However, it must be underlined that further types of errors can be observed in drawing-from-memory tasks where, for instance, some errors can arise from faulty access to semantic knowledge [21], particularly when drawing objects (e.g., Ref. [28]). For CDT, the most used drawing-to-command test, Libon et al. [29] proposed an operational error classification which can be summarized as follows: (i) graphomotor errors, which include abnormalities in the overall execution of the drawing such as alterations in clock size and imprecise/distorted clock face shape; (ii) errors in hand/number placement, including misplacement of hands, omission or incorrect sequencing of numbers, spatial disorganization, neglect of one side, and irregular spacing; (iii) executive control errors, such as counterclockwise numbering, perseverative behaviors, and inappropriate repetition of elements. Nonetheless, several other sets of criteria have been proposed, largely differing in their rationale and level of detail [14,30].

Several studies indeed ascribed the different types of visuo-constructional errors to alterations in specific cognitive domains. For instance, omissions of details have been found to correlate with attentional deficits or impaired global processing [31]. Graphic perseverations are often associated with executive and inhibitory control deficits [32]. Spatial distortions might reflect altered spatial representation [33], while rotations and inversions could indicate difficulties in spatial transformation processes [34]. Closing-in (CI) could be related to compensatory and/or attentional mechanisms [35,36,37].

Impairments in constructional abilities can be observed in virtually any neuropsychological condition [21] and have been frequently reported in neurodegenerative disorders. For example, in Alzheimer’s Disease (AD) a progressive impairment of constructional abilities emerges [21], with CI frequently observed in advanced stages [38]. In Frontotemporal Dementia (FTD) visuo-constructional impairments and altered planning are frequently reported and could be mainly ascribed to executive dysfunction [39,40]. In AD, deficits have been associated with temporo-parietal regions and the precuneus [41], whereas in FTD copying difficulties are linked to dorsolateral frontal dysfunction [42]. In patients with focal brain lesions, Chechlacz et al. [20] found that overall accuracy is correlated with right subcortical structures, while spatial errors are related to temporal and parietal lesions. However, the qualitative nature of the errors has received less attention, limiting full understanding of the specific cognitive mechanisms involved. For this reason, it seems worth scrutinizing the qualitative analysis of errors reported in the previous studies within the spectrum of dementia-related disorders. The present systematic review aims at synthesizing the literature on visuo-constructional errors in dementia to identify distinctive qualitative constructional patterns useful for characterizing different forms of dementia and to improve comprehension of the cognitive mechanisms involved in the genesis of constructional errors.

## 2. Methods

This systematic review was conducted according to PRISMA 2020 guidelines, and the completed PRISMA checklist is provided as Supplementary Material [43]. Although the review was not prospectively registered, predefined eligibility criteria and standardized data extraction procedures were applied to reduce potential biases.

### 2.1. Search Strategy

For selecting the relevant studies, a systematic search was performed on the databases PubMed, Scopus and Web of Science. The search included all studies between 1 January 1990 and 31 January 2026. The following search string was used and adapted to the specific databases: “(constructional apraxia OR constructive apraxia OR constructional abilit* OR constructive abilit* OR drawing task* OR drawing abilit* OR copying task OR visuoconstruct* OR constructive error*) AND (dementia OR neurodegenerative disorders OR cognitive impairment OR cognitive deterior* OR Alzheimer OR Fronto temporal dementia OR Frontotemporal dementia OR Parkinson OR lewy bod* OR Corticobasal dementia OR vascular dementia OR cognitive decline OR semantic dementia OR MCI OR primary progressive aphasia)” (see Supplementary Materials for database-specific search strings).

Additional articles were identified through the references cited in the reviewed papers.

### 2.2. Eligibility Criteria

Studies were considered eligible if they: (i) included adults (18+ years); (ii) included patients with a diagnosis of dementia or mild cognitive impairment (MCI) based on established criteria (e.g., DSM, ICD, Petersen criteria); (iii) assessed drawing or copying abilities with standardized or well described tasks; and (iv) provided qualitative assessment of errors. Exclusion criteria were: (i) articles not published in the English language in peer-reviewed journals; (ii) meta-analyses or reviews; (iii) single-case reports; and (iv) articles not explicitly assessing CA (e.g., using double task paradigms to modulate CA).

### 2.3. Study Selection Process

**Figure 1:**
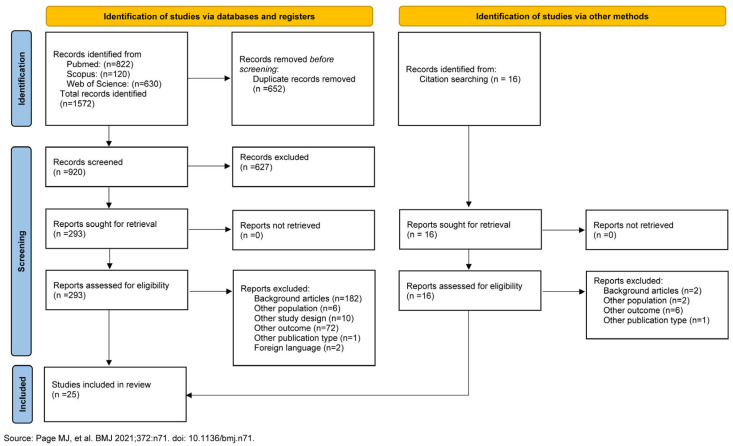
PRISMA 2020 flow diagram [44].

The process of study selection is described in the PRISMA diagram (Figure 1). The articles retrieved by the primary search were imported and screened for duplicates by an automated tool (Rayyan—https://www.rayyan.ai; accessed on 22 June 2026)—a web and mobile app for systematic review). Then articles were screened by title and abstract for relevance to the study variables. Finally, full-text studies were analyzed, and relevant information was extracted (see below). Included studies were then rated for quality of evidence. The selection and screening process as well as data extraction were performed by two independent reviewers (VC and FP); disagreements were resolved through discussion, and a third reviewer (LS) was consulted when consensus could not be reached. Quality assessment was performed by four independent reviewers (VC, FP, AF, AS) divided in 2 pairs and a further reviewer (LS) intervened in case of disagreement.

### 2.4. Quality Assessment

The quality of evidence was assessed using an adapted version of the Newcastle–Ottawa Scale (NOS; Refs. [45,46]) specifically tailored to the objectives of this review. Differently from the original version of the scale [45], for the purposes of this study some items and the relative score systems were adapted. In particular, the domain “clearness of the aim” was modified to consider the presence of clearly presented study purposes, hypotheses or both, independently of the number of cited studies. This adaptation was introduced because the literature specifically addressing visuo-constructional abilities in dementia is still limited. The “comparability” domain considered the presence of groups matched for demographic variables but did not consider differences in the sample size distribution, as sample size in neuropsychological populations largely depends on disease-specific epidemiological data. The item “statistical analysis” included the presence of a preliminary normality check on the data. This addition was considered relevant because the assessment of distributional assumptions is important for selecting appropriate statistical analyses, while this aspect was not explicitly covered in the original scale. A full description of each item is provided in the Supplementary Materials. Based on a star-scoring system, this version of NOS classifies studies as “high quality and low risk of bias” (13–16 stars), “moderate quality and moderate risk of bias” (9–12 stars), “low quality and high risk of bias” (5–8 stars) and “unsatisfactory” (4 or fewer stars).

### 2.5. Data Extraction

From articles selected for review, the following data were extracted: authors, sample characteristics, demographic information (age), drawing or copying task, study design, comparisons, main findings and quality.

### 2.6. Error Classification

To classify the errors reported in copying tasks, the seven categories borrowed from Critchley [2] were used: untidy execution, spatial misplacement/neglect-related errors, spatial-related errors, simplifications, rotation errors, perseverations and closing-in. It is worth mentioning that the proposed distinction between overlap-CI (when at least part of the copy intersects or overlaps the model) and near-CI (when the copy is produced in close proximity to the model), which could be related to differential mechanisms [47], will not be considered here because it is not widely adopted in the literature. A further category was added to consider altered copying procedures (planning deficits) in the studies assessing the order of drawn elements when copying the ROCF.

Since the types of errors reported in the included studies differed greatly and did not always conform to the proposed classification framework, two reviewers (FP and VC) independently assigned study-specific error types to one type of the present classification; any disagreement was resolved through discussion or, when necessary, by consulting a third reviewer (LT).

Errors observed in CDT to command (and to copy) were not classified within a coherent framework, as very heterogeneous scoring criteria are present in the literature, in most cases providing scores from which it is not possible to derive the number or the percentage of the different error types (e.g., Refs. [14,29,30]). It also has to be considered that, in CDT, it is difficult to disentangle proper visuo-constructional errors from those related to impaired access to conceptual knowledge [21]. Due to the heterogeneity in study designs, tasks, and qualitative scoring systems, no formal analyses of heterogeneity or sensitivity analyses were performed.

## 3. Results

At the end of the selection process, 25 studies entered the qualitative synthesis (Table 1). The involvement of the third reviewer to deal with disagreements during the selection process was required for only one article.

### 3.1. Quality Evaluation

The quality of the studies was judged to be “moderate” for two papers [48,49], “low” for nineteen studies [30,50,51,52,53,54,55,56,57,58,59,60,61,62,63,64,65,66,67] and “unsatisfactory” for four studies [68,69,70,71]. Most studies received the lowest rating because of poorly defined criteria for assessing sample size, poor sample representativeness and control of confounding factors, small sample sizes and lack of control for demographic factors (Table 2).

### 3.2. Study Characteristics

The 25 studies included in the review were analyzed for the experimental design adopted, the neurodegenerative disorders considered, and the task used to assess CA (Table 1).

Twelve studies were cross-sectional [30,49,51,56,58,59,63,65,66,69,70,71], eight were retrospective [55,57,60,61,62,64,67,68] and five were longitudinal prospective studies [48,50,52,53,54]. Most studies enrolled patients with different diagnoses, so cumulating data across the studies, nineteen papers reported constructional errors in AD [30,48,49,50,51,52,54,55,56,57,58,59,62,64,65,66,67,69,71], three in MCI-AD [60,61,70], two in MCI-DLB [60,61], five in FTD [49,51,52,64,65], three in DLB [49,54,57], five in MCI (both amnesic and non-amnesic, both single- and multiple-domain; refs. [58,63,66,68,70]), five in VD [48,49,56,59,62], one in MCI-VD [70], two in HD [30,55], three in PDD [49,52,59]. In fourteen studies, performance of the clinical group(s) was compared with that of a group of matched healthy controls (HC).

**Table 1:** Methodological and demographical aspects of the studies included in this review.

First Author	Participants	Age	Drawing or Copy Task	Study Design	Comparisons	Main Findings
Ahmed [63]	51 mMCI, 22 dMCI, 13 aMCI, 43 HC	71.29 (9.36), 74.60 (9.73), 69.53 (8.53), 73.07 (7.42)	CDT both on command and copy	Cross-sectional	ANOVAs to assess group differences in CDT performance. Mann–Whitney tests to compare distribution of errors	More errors involving hand/number placement in dMCI and mMCI than in aMCI and HC
Ambron [68]	15 aMCI, 12 non-aMCI, 98 m-aMCI, 29 m-non-aMCI	NA	Copy of ROCF	Retrospective cross-sectional	Chi square test	CI more frequent in mMCI than in sMCI
Beretta [57]	35 DLB, 25 AD	75.09 (6.56), 65.85 (6.51)	Copy of Pentagons	Retrospective cross-sectional	Two-sample *t*-test	Rotations more frequent in DLB than in AD
Cagnin [60]	25 MCI-DLB, 28 MCI-DLB	76.5 (4.9), 73.4 (6.7)	Copy of Pentagons	Retrospective cross-sectional	*t* test for independent groups and Mann–Whitney U test; Chi-square test for categorical variables	Number of angles significantly lower in DLB than in AD; no difference for the total score of Pentagons and remaining subscores
Cagnin [61]	30 MCI-DLB, 23 MCI-AD	75.4 (6.2), 72.6 (6)	Copy of Pentagons	Retrospective cross-sectional	*t* test for independent groups; Chi Square tests	No difference in the total score of Pentagons between groups; lower number of angles in MCI-DLB than in MCI-AD
De Lucia [62]	114 AD; 63 VD	74.91 (6.8), 76.4 (6.4)	CAT	Retrospective cross-sectional	MANOVA on total number of perseverations, number of recurrent perseverations, and number of continuous perseverations with AD and VD and mild vs. moderate-severe severity as between-subject factors	In both AD and VD, individuals with moderate-to-severe dementia produced significantly higher numbers of total perseverative errors, recurrent perseverations and continuous perseverations, than people with mild dementia
Di Cecca [55]	41 HD, 25 AD, 35 HC	50.68 (14.43), 71.24 (6.47), 53.09 (12.82)	CAT, ROCF copy	Retrospective cross-sectional	ANOVA with group as between subject factor on number of each type of errors	CI more frequent in AD than in HD and healthy individuals; distortions more frequent in HD than in AD; rotations, omissions, transpositions and simplification more frequent in than in HD
Dion [53]	173 HC, 28 MCI	68.50 (5.80), 70.60 (8.12)	CDT both on command and copy	Longitudinal, cross-sectional	ANCOVA with groups as a between subject factor and demographic variables as covariates	Larger digit misplacement in both command (43°) and copy (19°) condition in MCI
Duro [49]	242 AD, 112 FTD, 130 VD, 59 DLB, 70 PDD, 300 HC	74.49 (7.56), 67.53 (8.73), 69.98 (9.91), 76.1 (6.47), 69.74 (10.23), 68.91 (6.91)	CDT only on command	Retrospective	Chi-square test for qualitative constructional errors	Stimulus-bound responses more frequent in AD than in HC, VD and PDD; conceptual errors more common in AD and DLB
Freeman [59]	26 AD, 25 IVD, 12 PDD, 20 HC	77.8 (6.3), 77.5 (6.2), 76.4 (7.5), 75.3 (7.1)	Copy of a modified version of the ROCF	Cross-sectional study	MANCOVAs investigating between-group differences on configuration, cluster, fragmentation, placement omission and perseveration errors	HC outperformed all clinical samples; fragmentations, misplacements and omissions more frequent in PDD and IVD than in AD; perseverations more frequent in PDD than in AD and IVD
Gasparini [64]	15 mild FTD-bv, 41 mild AD	65 (4.72), 67.8 (4.88)	ROCF copy	Retrospective cross-sectional	Chi-square test with odds ratio	Similar ROFC scores, execution strategies, types of error and global analysis in AD and FTD; impairment in inner details production more frequent in FTD than in AD
Grossi [65]	14 bv-FTD, 11 AD, 64 HC	67.2 (5.4), 66.7 (7.6), 64.2 (7.7)	Simple geometrical drawings (set C)	Cross-sectional	Chi-square test for qualitative constructional errors	Spatial distortions were the most frequent type of error both in bv-FTD and AD, with no differences between the two groups
Kwak [56]	30 healthy young, 22 healthy elderly, 98 AD, 48 SVD	29.48 (5.90), 67.30 (8.46), 76.92 (8.03), 76.75 (5.87)	Copy of Luria’s lines	Cross-sectional	Chi-square test	CI more frequent in AD than in SVD
Lazarova [67]	384 AD, 512 HC	74.78 (7.94), 73.44 (6.28)	CDT both on command and copy	Cross-sectional	Chi-square test for absence or presence of constructional errors	Lower scores and higher error rates in AD than in HC
Lee [52]	94 AD, 119 PDD, 22 VD	71.76 (6.71), 70.09 (8.10), 71.95 (7.62)	CDT only on command	Longitudinal, cross-sectional	Multiple response analysis with dementia subtypes or level of dementia as between-subject factor on error frequency	Longitudinal changes on CDT did not differ among dementia subtypes; many spatial and planning errors in mild to moderate stages; predominant conceptual errors in severe dementia
Lima [51]	18 bv-FTD, 18 AD	62.56 (8.26), 56.29 (6.41)	Cube copy	Cross-sectional	Non-parametric Kruskal–Wallis test analysis for group comparisons	Lack of elements, CI and gestalt changes more frequent in AD; perseveration, lack of planning and inattention more frequent in bvFTD
Lo Buono [48]	50 VD, 50 AD	71.14 (7.75), 74.03 (7.71)	Copy of Pentagons	Longitudinal, cross-sectional	Wilcoxon signed-rank test for intragroup analysis (T0 vs. T1); Mann–Whitney U test for intergroup analysis	All types of errors (number of angles, rotation, closure opening, intersection) more frequent in VD than in AD at both T0 and T1
Malloy [58]	43 HC, 43 MCI, 40 AD	73.33 (6.1), 74.77 (8.7), 76.65 (6.2)	Beery’s Developmental Test of Visual-Motor Integration	Cross-sectional study	Between-group ANOVA and ROC curve	Higher scores in HC than both MCI and AD, and in MCI than in AD; more errors in AD than in MCI and HC; stimulus-bound errors and few perseverations found in both MCI and AD
Mitolo [54]	16 DLB, 15 AD	78.67 (6.76), 83.13 (5.74)	Copy of Pentagons	Longitudinal, cross-sectional	Independent *t* tests for each type of errors; repeated measure ANOVAs on types of errors with group as between subject factor and time evaluation as within subject variable	At the first evaluation lower number of angles in DLB than in AD; 1 year before death more rotation in DLB than in AD; steeper decline in DLB than in AD
Parsey [66]	33 AD, 33 MCI, 66 HC	74.06 (6.62), 70.52 (9.42), 71.92 (8.75)	CDT only on command	Cross-sectional	Chi-square analyses to assess significant group differences	Higher number of conceptual errors and graphic difficulties in MCI than in HC; more perseverations, graphic errors, conceptual, spatial and stimulus-bound errors in AD than in MCI
Rouleau [30]	25 AD, 25 HD, 25 HC	71.00 (6.70), 49.84 (12.78), 70.88 (6.65),	CDT both on command and copy	Cross-sectional	Chi square on groups and types of errors	Larger clock size, more conceptual errors and more perseverations in AD than in HD
Salvadori [70]	30 degenerative MCI, 27 vascular MCI	75.2 (4.4), 73.2 (6.9)	ROCF copy	Cross-sectional	Independent sample *t* tests	Lower number of fragmentation and perseveration in Vascular than in degenerative MCI
Toukko [69]	62 HC, 58 AD	71.3 (8.07), 70.6 (7.45)	CDT only on command	Cross-sectional study	MANCOVAs controlling for education to compare the groups on drawing, setting and reading the clock. Chi-square tests to compare the samples for error types.	The groups differed on all three measures; more omissions in number and hands drawing and more misplacements in AD than in HC
Wang [50]	45 AD (converters), 76 HC (non converters)	69.4 (6.8), 68.8 (9)	CDT only on command	Longitudinal, cross-sectional	*t*-test (converters vs. non converters)	Not equally spaced numbers, absence of at least one number, numbers not placed clockwise, variable distance between numbers, clock with less or more than two hands, missing arrows more frequent in AD than in HC
Watson [71]	40 AD, 36 HC	NA	CDT only on command	Cross-sectional	kappa value for each variable	Errors in the upper left quadrant of clock have the best specificity and sensitivity to identify dementia

Notes. AD = Alzheimer Disease; FTD = Fronto-Temporal Dementia; VD = Vascular Dementia; SVD = Small Vessel Dementia; IVD = Ischemic Vascular Dementia; HD = Huntington Disease; PDD = Parkinson Disease with Dementia; MCI = Mild Cognitive Impairment; dMCI = Dysexecutive MCI; aMCI = Amnesic MCI; m-aMCI = Multi-domain aMCI; non-aMCI = Non-Amnesic MCI; m-non-aMCI = Multi-domain non-aMCI; mMCI = Multi-domain MCI; sMCI = Single-domain MCI; DLB = Dementia of Lewy Bodies; bv = behavioral variant; HC = healthy controls; CDT = Clock Drawing Test; ROCF = Rey–Osterrieth Complex Figure; CAT = Constructional Apraxia Test; CI = Closing-In; NA = not provided.

**Table 2:** Quality evidence of the studies assessed by modified version of Newcastle–Ottawa Scale (NOS).

First Author	Aim (0–2)	Sample Selection (0–8)				Comparability (0–2)		Outcome (0–4)		Total (0–16)
		Representativeness (0–2)	Sample Size (0–2)	Non-Response Rate (0–2)	Exposure Assessment (0–2)	Control of Confounding Factors (0–1)	Comparability of Groups (0–1)	Assessment of the Outcome (0–2)	Statistics (0–2)	
Ahmed [63]	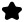 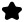				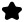 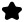				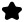	5
Ambron [68]					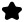 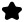			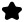	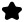	4
Beretta [57]	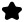 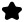			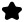	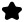 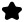			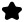 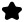	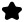	8
Cagnin [60]	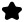				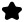 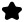		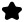	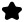	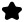	6
Cagnin [61]	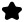				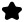 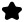		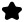	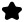 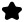	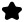 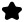	8
De Lucia [62]	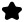 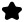				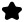 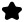				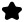 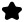	6
Di Cecca [55]	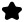 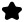		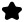 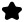	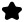	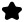 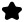				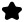	8
Dion [53]	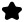 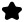			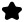	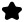 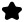				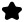 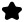	7
Duro [49]	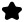		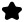		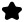 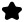	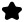	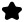	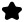 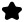	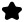	9
Freeman [59]	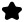 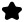			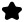	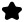				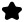	5
Gasparini [64]	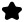			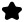	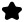 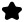		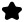	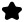	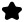	7
Grossi [65]	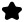			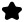	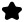 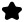			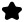	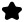	6
Kwak [56]	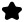			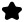	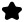 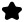		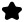			5
Lazarova [67]	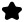		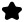	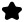	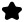	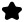			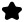	6
Lee [52]	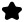				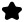 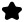			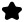 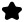	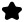	6
Lima [51]	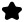			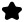	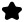		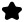	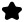 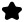	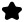 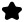	8
Lo Buono [48]	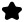				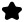 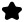	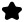	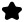	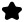 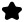	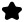 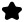	9
Malloy [58]	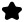 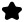				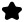 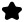				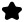	5
Mitolo [54]	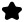				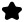 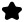		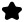		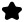	5
Parsey [66]	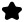 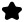				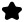 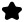		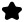	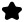 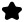	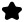	8
Rouleau [30]	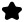				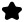			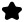 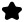	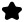	5
Salvadori [70]	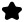				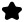 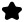				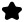	4
Toukko [69]					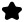 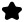		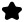		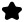	4
Wang [50]	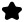				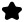 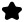				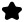 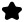	5
Watson [71]	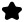				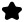			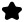		3

NOS scoring system. 13–16 stars: high quality and low risk of bias; 9–12 stars: moderate quality and moderate risk of bias; 5–8 stars: low quality and high risk of bias; 4 or fewer stars: unsatisfactory.

In terms of the constructional tasks used, fifteen studies employed copying tasks: four used the standard version of the ROCF [55,64,68,70], one used a modified version of the ROCF [59], four used two interlaced pentagons [48,54,57,60,61], two used the CAT [55,62], one assessed cube copying [51], one used copying of Luria’s lines [56], one used Beery VMI [58], and one used set C of Benton’s Visual Retention test [65]. Of these studies, only three [54,56,62] examined constructional errors in relation to dementia severity.

Ten studies employed the CDT, i.e., they required drawing a clock to verbal command (refs. [30,49,50,52,53,63,66,67,69,71]), and four of them also employed a clock copying condition [30,53,63,67]. Only two studies [52,71] examined CDT performance in relation to dementia severity.

### 3.3. Description of the Findings

Among the 25 studies selected for the present review, only four reported a quantification of the different types of errors in copying tasks, two employing the ROCF [59,64], one employing copying of geometrical figures [65], and one employing both [55], to allow characterizing error types in different dementia conditions, and comparing such features between the conditions. A further four studies assessed the copy of two interlaced pentagons [48,54,57,61], for which a quantitative scoring of different types of errors was provided [72]. According to this method, the authors assigned specific scores to: (i) overall number of angles drawn, (ii) presence of intersection between the two figures, (iii) presence of appropriately closed figures, (iv) rotation of figures, and (v) closing-in. While the last two categories can be approximated to the corresponding types of errors borrowed from Critchley (Table 3), the remaining three might include both spatial errors and simplifications.

In six studies employing the CDT [30,49,50,66,67,69], error types were scored according to very heterogeneous criteria and level of detail (Table 4).

The remaining ten studies did not provide a quantification of error types. Some of them cumulated different error types within a cumulative score [58,60,63], or provided a sketchy description of the most frequent errors in their sample [51,52], whereas five studies only focused on one specific error type, i.e., perseverations [62], closing-in [56,68], an altered procedure in copying the ROCF [70] or angular displacement [53] and number of digits in each quadrant [71] in CDT.

In the following paragraphs, the errors reported in the copying tasks and in the CDT will be described separately.

### 3.4. Copying Tasks

#### 3.4.1. Untidy Execution

No studies considered errors related to basic motor impairments, which were in some studies explicitly ignored (e.g., Refs. [54,60,61]).

#### 3.4.2. Spatial Misplacement/Neglect-Related Errors

No studies included in this review reported lateralized constructional errors related to spatial neglect.

#### 3.4.3. Spatial-Related Errors

In the studies providing a quantification of errors, those related to spatial configuration were those most frequently reported in most conditions (i.e., AD, FTD, HD, VD, PDD). Nonetheless, considering the studies reporting direct comparisons between single neurodegenerative conditions, some distinctive patterns emerged.

Spatial distortion errors were comparable between AD and FTD [65], whereas they were more frequent in HD compared to both AD and HC [55].

Spatial misplacement errors were less frequent in AD than in PDD and VD [59].

#### 3.4.4. Rotation Errors

Rotation errors were described in many studies, although at a quite low rate and with variable distribution across clinical groups. AD and FTD patients showed similar performance [65], whereas more rotation errors were found in DLB than in AD, particularly at later stages [54,57]. Longitudinal findings indicated an increase in rotation errors over time in both AD and VD [48]. By contrast, no differences emerged between AD and HD [48]. However, transposition errors were more frequent in AD compared to HD and HC [55].

#### 3.4.5. Perseveration

Perseverative behaviors have been reported in many studies but at low rates and did not consistently distinguish between diagnostic groups. Similar frequencies were reported in AD and VD [62], as well as between AD and HD [55]. Higher rates were found in PDD compared to both AD and VD [59]. In earlier disease stages, perseverations were less frequent in MCI-VD than in MCI-AD [70].

#### 3.4.6. Simplification

These errors represented the second most prominent feature across studies, and their expression varied depending on the clinical condition. Simplification errors overall were more frequent in AD compared to HD and HC [55]. Impairment in drawing inner details of complex stimuli was greater in FTD than in AD [64]. Fragmentation errors also showed variability across groups: these were lower in MCI-VD compared to MCI-AD [70], whereas fragmentation errors were less frequent in AD than in PDD and VD [59]. Non-lateralized omissions followed a differential pattern, being more frequent in AD compared to FTD and HD [51,55], as well as compared to HC [55]. Conversely, omissions were fewer in AD than in PDD and VD [59].

Fewer angles were observed in MCI-DLB compared to MCI-AD [60,61] and, similarly, in DLB compared to AD [54], with longitudinal evidence pointing to a progressive decline in VD [48]. Closure difficulties were more frequent in VD compared to AD [48] and in DLB compared to AD [54].

#### 3.4.7. Closing-In

The closing-in phenomenon was consistently reported across studies. It was more frequent in multi-domain non-amnesic MCI than in amnesic MCI [68] and more common in AD compared to MCI [58]. Moreover, AD patients exhibited higher rates of CI than those with FTD [51], SVD [56], and HD [55].

#### 3.4.8. Altered Drawing Procedure (Planning Deficits)

All the studies addressing copying procedures reported difficulties related to planning and strategy execution in copying the ROCF. Greater impairment in initiation and execution processes was observed in bvFTD than in AD [51]. In addition, alterations in the organization of drawing strategies were observed, reflecting inefficient constructional approaches in AD and FTD [64], and in about 65% of patients with MCI [68]. However, no significant differences between AD and FTD were found in the type of strategy adopted during copying, with both groups predominantly relying on piecemeal approaches rather than global strategies [64].

### 3.5. Clock Drawing Test

Changes in clock size were consistently reported across studies. Patients with AD tended to produce larger clocks than patients with HD [30] or MCI [66], and than HC [66,67], whereas longitudinal findings indicated a reduction in clock size in AD compared to MCI over time [50]. Other graphic errors were described in AD and MCI [66,67].

In the command condition, patients with multi-domain-MCI and dysexecutive-MCI committed more errors in hand and number placement than amnesic-MCI and HC [63]. Analogously, both in command and in copy, patients with AD committed more errors in number and hand placement than HC [67]. Digit misplacement errors were more frequent in MCI than in HC [53]. Spatial layout errors were more common in PDD and VD compared to AD [52] and less frequent in MCI and HC [66]. Duro et al. [49] found that the same kind of errors was more frequent in AD, VD and DLB than in HC, and that more errors were present in DLB than in FTD, VD and PDD. Omissions of numbers and hands were also more frequent in AD compared to both HC and MCI [50,69] and a reduced number of digits in the fourth quadrant has been proposed to be specific to AD [71].

Several error types could reflect executive control impairments according to Libon et al. [29]. Non-clockwise drawing was observed in AD and increased with disease progression [49,50], while perseverations were reported as part of executive dysfunction [50] and were more frequent in AD and DLB than in FTD, MCI and HC [49,66]. In addition, stimulus-bound responses were more frequent in AD, FTD, VD, DLB and PDD than in HC [49], and in AD showed more frequently than in VD, PDD and MCI [49,66]. Regarding planning deficits, Lee et al. [52] found that they were more evident in PDD and VD compared to AD, while Duro et al. [49] did not observe differences among AD, FTD, VD and PDD, but all groups committed more planning deficits than HC.

Finally, more conceptual errors were reported in AD than in PDD and VD [49,52], FTD [49], MCI [66] and, similarly, in HD [30], whereas conceptual errors were more frequent in DLB than in HC and FTD [49].

## 4. Discussion

This systematic review analyzed the qualitative patterns of visuo-constructional errors in neurodegenerative conditions, with the aim of identifying the regularities and possible peculiarities of errors in each type of dementia. The qualitative synthesis of the selected studies showed that in copying tasks, no constructional errors attributable to neglect were reported in any of the included studies, while untidy execution related to motor disturbances was not reported or systematically disregarded. The most common error categories reported across the studies were spatial errors and simplifications, whereas perseverations, rotations and closing-in were very often reported, but at a lower rate.

However, the quality of most of the studies included in the review was rated as low or unsatisfactory; thus, the results should be interpreted with caution. Moreover, a limited number of studies addressed the specific influence of dementia severity on constructional errors; thus, no firm conclusions can be drawn on this issue.

The following sections will describe the cognitive profiles associated with constructional errors in different dementia conditions and provide an overview of the contrasts observed across disorders.

### 4.1. Cognitive Profile of Constructional Errors in Dementia (And Their Neural Correlates)

The finding of CA across conditions confirmed that impairments in multiple cognitive processes can generate visuo-constructional deficits. In AD several elements of the model could often not be integrated with each other, leading to spatial-related errors [30], with a progressive worsening of errors as the disease progresses [21]. In addition, on the CDT, patients with AD frequently show omissions and conceptual errors, together with alterations in clock size [49,50,52,66,67,69,71]. The present picture probably reflects deficits in visuospatial integration, global representation and conceptual processing associated with temporo-parietal involvement in Alzheimer’s disease [41,74,75]. In FTD, the difficulties in planning deficits (strategy/execution) and sequencing of actions could represent an issue for the constructional tasks, whereas spatial abilities could remain relatively spared [42,76], suggesting a primary executive dysfunction related to frontal involvement [40]. Executive-related errors such as stimulus-bound responses and perseverations have also been reported in these patients, possibly reflecting impaired inhibitory control and cognitive flexibility [49,50,66]. In DLB and PDD, drawing performance is frequently characterized by spatial-related errors [52,57], which can be ascribed to alterations in posterior visual systems, attentional circuits and occipital hypometabolism that characterize these neurodegenerative conditions [77,78,79,80]. Deficits of executive function and spatial layout have also been described, indicating the involvement of these processes [49,52]. In HD, simplifications, perseverations and planning deficits (strategy/execution) have been often observed [30,31,32,81], reflecting dysfunction in fronto-striatal circuits and the contribution of reduced processing speed and motor impairment [82,83]. In VD, the constructional profile is not homogenous, with prominent spatial-related errors and rotation errors [33,34,84], likely in relation to larger variability in lesion site and white matter damage [85] with respect to the other dementing conditions.

In MCI, qualitative characteristics may reveal early neural alterations: visuo-constructional deficits in amnesic-MCI are generally less pronounced and secondary to mnesic dysfunction [86,87], whereas spatial-related errors and planning deficits (strategy/execution) in non-amnesic-MCI and multi-domain-MCI suggest early impairment of executive and visuospatial processes [88,89,90,91]. Spatial errors have also been observed, indicating early disruption of spatial organization and executive processes [53,63]. A specific defect has often been reported in SD, where the impairment of semantic processing might generate over-simplified and incorrect feature attribution in instances of drawing from memory [28,92,93].

### 4.2. Differences in Qualitative Error Categories Between AD and Other Dementia Conditions

The aim of this systematic review was to examine the potential contribution of qualitative analysis of constructional errors in characterizing dementia syndromes. However, a limited number of studies directly compared different dementia syndromes in terms of qualitative constructional errors, so it is not possible to address this issue across multiple conditions. This limitation is further compounded by the lack of a shared and standardized classification of constructional errors across studies. Indeed, most investigations adopt only partially overlapping or non-equivalent frameworks, which often prevents the direct alignment and comparison of error categories across studies. For these reasons, the present findings allow only a broad distinction between AD and other forms of dementia. AD is characterized by a prominent involvement of spatial-related errors, reflecting impaired global organization of the drawing, whereas these alterations appear more strongly related to visuoperceptual and spatial disorganization in conditions such as PDD and VD [52,59]. Simplifications in AD tend to reflect a reduction in structural complexity, while in FTD they are more often associated with inefficient constructional strategies and reduced generation of internal details [55,64]. Regarding omissions, AD shows a higher frequency of missing elements compared to FTD and HD, suggesting a deficit in integrating the overall configuration, whereas in PDD and VD omissions are less consistently expressed and may be accompanied by broader spatial disorganization [51,55,59]. The closing-in phenomenon has been described in virtually all conditions but is more frequent in AD than in FTD, VD and HD, supporting the hypothesis that in AD it could reflect a reduced ability to build and maintain an internal representation of the model associated with defects in attentional control [51,55,56,58]. Executive-related errors are more prominent in FTD, PDD and HD compared to AD, reflecting a greater impairment in action monitoring and control, whereas in AD these features tend to emerge with disease progression [49,50,59,64,83]. Finally, conceptual errors are more consistently associated with AD compared to other dementias, indicating a more frequent disruption of semantic knowledge underlying the constructional task [30,49,52,66].

### 4.3. Limitations and Future Directions

The qualitative analysis of constructional errors in neurodegenerative diseases is still under-explored and requires further efforts. Given the methodological issues highlighted above, future research should aim to adopt a shared and standardized categorization of constructional errors. The use of established frameworks, referring to Critchley’s classification [2] for copying tasks as in the present review, or to Libon’s criteria [29] for the Clock Drawing Test, would improve consistency across studies. This would allow more reliable comparisons between different dementia conditions and facilitate the identification of qualitative markers.

An important finding that emerges consistently across studies is that the diagnostic significance of errors also depends heavily on the type of task used. Copying tasks allow us to assess some aspects of visuo-spatial and visuo-constructional abilities but are strongly dependent on the complexity and the specific features of the items. For instance, the copy of two interlaced pentagons led to the observation of errors, such as reduced number of angles, missing intersections, and rotations, whose potential contribution to the characterization of different neurodegenerative diseases has been repeatedly explored [48,57,72]. The ROCF, on the other hand, appears to be more informative for capturing aspects related to copying strategy, fragmentation, simplification and overall spatial organization [30,64,70]. Instead, drawing from memory tasks, such as the CDT, appear to be particularly sensitive to errors involving executive and semantic components, such as the incorrect positioning of numbers, the misalignment of clock hands or the loss of the concept of a clock [14,50,52]. These observations should inform the choice of evaluation tools, some of which allow for the identification of specific qualitative errors, but the heterogeneity of tasks and scoring systems does not help comprehend differential features of visuo-constructional impairments in neurodegenerative disorders [21].

It is important to underline that more efforts should be made to overcome the limitations identified in the selected studies related to small sample sizes, to poor sample representativeness and to the control of confounding factors.

Future research should aim to develop and adopt consistent operational description of error types to improve comparability across studies and enable quantitative analyses of error frequency across dementia conditions. Moreover, future studies should integrate qualitative analyses with neuroimaging data and adopt longitudinal designs to assess the prognostic value of error patterns [20,23]. It would also be useful to link the type of error to its impaired neural correlates to contribute not only from a qualitative perspective but also in terms of the neural circuits involved, thereby strengthening the diagnostic value of constructional errors.

A further set of limitations is related to the methodology followed by the present systematic review. Although the lack of prospective protocol registration represents a limitation, potential sources of bias were controlled by strictly adhering to the PRISMA 2020 guidelines. This included the use of predefined eligibility criteria, a structured search strategy, and independent screening and data extraction procedures. Moreover, the inclusion was restricted to studies published in English to ensure linguistic consistency during screening, data extraction, and interpretation. This choice, however, could have led to overlooking relevant studies published in other languages.

## 5. Conclusions

Many of the studies selected for the present review suggested that qualitative differences between clinical conditions exist and often pointed to one specific feature or to a combination of features as possible behavioral indicators of the different conditions. Nonetheless, there is also a significant overlap between neurodegenerative diseases. Errors such as omissions, perseverations, simplifications or distortions can be observed in several neurodegenerative conditions, albeit at different rates. No single error, considered in isolation, therefore appears to be pathognomonic of a specific form of dementia. For example, perseverations may occur in FTDs, but also in AD, VD and PDD [52,59,62]; omissions often characterize AD but are also present in other disorders such as VD; rotations are particularly prominent in DLB but not entirely absent elsewhere. This means that clinical interpretation should not be based on the identification of a single distinctive sign but rather integrated with the overall neuropsychological profile and the temporal course of the condition. In conclusion, the present review suggests that the qualitative analysis of visuo-constructional errors could offer potential added value for the clinical description of dementia and, possibly, for monitoring its therapeutic management [94], but further efforts are needed in this field.

## Figures and Tables

**Table 3:** Correspondence of constructional error categories across studies.

Constructional Errors Classification (Adapted from Critchley)	Error Types Described in Each Study
Untidy execution	Not reported (explicitly excluded in [54,60,61])
Spatial misplacement/neglect-related errors	Not reported
Spatial related errors	Distortion [55,65], Misplacement [59] Transposition [55]
Rotation errors	Rotations [48,54,55,57,65]
Perseveration	Perseveration [55,62,70]
Simplification	Simplification [55] Omission [55,59] Lack of inner details [64] or elements [51] Fragmentation [59,70] Number of angles [54,60,61] Closure of figure impairment [48,54]
Closing-in	Closing-in [51,56,58,68]
Planning deficits	Initiation and execution processes [51] Execution strategies [64,68]

**Table 4:** Criteria used to classify CDT errors across studies.

First Author	Criteria Used for CDT Error Analysis	Dimensions Considered
Ahmed [63]	Rouleau scoring system [30], adapted by Libon et al. [29]	10 error items per condition: command and copy scored separately; individual errors coded as present/absent. The article refers to Libon et al. [29] for the full item list.
Dion [53]	Digital Clock Drawing Test	1 digital metric: digit misplacement, calculated as deviation from the ideal angular position of each digit.
Duro [49]	Cahn scoring system [73]	8 dimensions: stimulus-bound response; conceptual deficit; perseveration; left hemispace neglect; planning deficit; nonspecific spatial error; numbers outside clock; numbers counterclockwise.
Lazarova [67]	Classification developed by the authors	10 binary variables: draw circle; clock symmetry; clock numbers; clock hands; clock time; copy circle; copy symmetry; copy numbers; copy hands; copy time.
Lee [52]	Rouleau qualitative criteria for error types [30]	4 dimensions: stimulus-bound response; conceptual deficit; spatial and/or planning deficit; perseveration error.
Parsey [66]	Modified Rouleau method [30]	6 dimensions: size of the clock; graphic difficulties; stimulus-bound responses; conceptual deficits; spatial and/or planning deficits; perseveration.
Rouleau [30]	Classification developed by the authors	6 dimensions: size of the clock; graphic difficulties; stimulus-bound response; conceptual deficit; spatial and/or planning deficit; perseveration.
Tuokko [69]	Classification developed by the authors	7 dimensions: perseverations; omissions; rotations; misplacements; distortions; substitutions; additions.
Wang [50]	Classification developed by the authors	7 final items: numbers equally spaced; other eight numbers marked; numbers clockwise; all numbers correct; constant distance between numbers; two hands; arrows drawn.
Watson [71]	Authors’ own objective Clock Completion Test classification	4 quadrant-based dimensions: first quadrant; second quadrant; third quadrant; fourth quadrant, with greatest weight assigned to errors in the fourth quadrant.

## Data Availability

No new data were created or analyzed in this study.

## Supplementary Materials

The following supporting information can be downloaded at https://www.mdpi.com/article/10.3390/brainsci16070667/s1. Table S1: PRISMA 2020 for Abstracts Checklist; Table S2: PRISMA 2020 Checklist; Text S1: Database-specific search strings and Scoring system of the modified NOS scale.

## Abbreviations

The following abbreviations are used in this manuscript:

AD	Alzheimer Disease
FTD	Fronto-Temporal Dementia
VD	Vascular Dementia
SVD	Small Vessel Dementia
IVD	Ischemic Vascular Dementia
HD	Huntington Disease
PDD	Parkinson Disease with Dementia
MCI	Mild Cognitive Impairment
dMCI	Dysexecutive MCI
mMCI	Multi-domain MCI
sMCI	Single-domain MCI
aMCI	Amnesic MCI
DLB	Dementia of Lewi Body
bvFTD	Behavioral variant of Fronto-Temporal Dementia
HC	Healthy controls
CDT	Clock Drawing Test
ROCF	Rey–Osterrieth Complex Figure
CAT	Constructional Apraxia Test
CI	Closing-In
NA	not available
