# Supplementary material for: Qualitative Analysis of Constructional Errors in Neurodegenerative Conditions: A Systematic Review
Source: Brain Sci. 2026 Jun 25;16(7):667. doi: 10.3390/brainsci16070667 (PMC13407041; doi:10.3390/brainsci16070667)
Supplement: Supplementary file 1 [file brainsci-16-00667-s001.zip › Text S1. Search strings_NOS Scale.pdf]

Supplementary Materials

Qualitative analysis of constructional errors in neurodegenerative conditions: a systematic review

Vincenzo Crisci, Laura Sagliano, Antonella Ferrara, Alessia Salzillo, Luigi Trojano, Francesco Panico

Department of Psychology, University of Campania "Luigi Vanvitelli", Viale Ellittico 31, 81100,

Caserta, Italy

Database-specific search strings

Pubmed

((("constructional apraxia"[Title/Abstract]) OR ("constructive apraxia"[Title/Abstract]) OR ("constructional abilit*"[Title/Abstract]) OR ("constructive abilit*"[Title/Abstract]) OR ("drawing task*"[Title/Abstract]) OR ("drawing abilit*"[Title/Abstract]) OR ("copying task"[Title/Abstract]) OR ("visuoconstruct*"[Title/Abstract]) OR ("constructive error*"[Title/Abstract])) AND (("dementia"[Title/Abstract]) OR ("neurodegenerative disorders"[Title/Abstract]) OR ("cognitive impairment"[Title/Abstract]) OR ("cognitive deterior*"[Title/Abstract]) OR ("Alzheimer"[Title/Abstract]) OR ("Fronto temporal dementia"[Title/Abstract]) OR ("Frontotemporal dementia"[Title/Abstract]) OR ("Parkinson"[Title/Abstract]) OR ("lewy bod*"[Title/Abstract]) OR ("Corticobasal dementia"[Title/Abstract]) OR ("vascular dementia"[Title/Abstract]) OR ("cognitive decline"[Title/Abstract]) OR ("semantic dementia"[Title/Abstract]) OR ("MCI"[Title/Abstract]) OR ("primary progressive aphasia"[Title/Abstract])))

SCOPUS

TITLE-ABS-KEY ("constructional apraxia" OR "constructive apraxia" OR "constructive abilit*" OR "constructional abilit*" OR"drawing task*"OR"drawing abilit*" OR "copying task" OR "visuoconstruct*" OR "constructive error*") AND TITLE-ABS-KEY ("dementia" OR "neurodegenerative disorders" OR "cognitive impairment" OR "cognitive deterior*" OR "Alzheimer" OR "Fronto temporal dementia" OR "Frontotemporal dementia" OR "Parkinson" OR "lewy bod*" OR "Corticobasal dementia" OR "vascular dementia" OR "cognitive decline" "semantic dementia" OR "MCI" OR "primary progressive aphasia")

WEB OF SCIENCE

(((TI=("constructional apraxia")) OR (TI=("constructive apraxia")) OR (TI=("constructional abilit*")) OR (TI=("constructive abilit*")) OR (TI=("drawing task*")) OR (TI=("drawing abilit*")) OR (TI=("copying task")) OR (TI=(visuoconstruct*)) OR (TI=("constructive error*")) OR (AB=("constructional apraxia")) OR (AB=("constructive apraxia")) OR (AB=("constructional abilit*")) OR (AB=("constructive abilit*")) OR (AB=("drawing task*")) OR (AB=("drawing abilit*")) OR (AB=("copying task")) OR (AB=("visuoconstruct*")) OR (AB=("constructive error*"))) AND ((TI=("dementia")) OR (TI=("neurodegenerative disorders")) OR (TI=("cognitive impairment")) OR (TI=("cognitive deterior*")) OR (TI=("Alzheimer")) OR (TI=("Fronto temporal

dementia")) OR (TI=("Frontotemporal dementia")) OR (TI=("Parkinson")) OR (TI=("lewy bod*")) OR (TI=("corticobasal dementia")) OR (TI=("vascular dementia")) OR (TI=("cognitive decline")) OR (TI=("semantic dementia")) OR (TI=("primary progressive aphasia")) OR (TI=("cognitive decline")) OR (AB=("dementia")) OR (AB=("neurodegenerative disorders")) OR (AB=("MCI")) OR (AB=("cognitive deterior*")) OR (AB=("Alzheimer")) OR (AB=("Fronto temporal dementia")) OR (AB=("Frontotemporal dementia")) OR (AB=("Parkinson")) OR (AB=("lewy bod*")) OR (AB=("corticobasal dementia")) OR (AB=("vascular dementia")) OR (AB=("dementia")) OR (AB=("neurodegenerative disorders")) OR (AB=("MCI")) OR (AB=("cognitive decline"))))

Scoring system of the modified NOS scale

Clearness of the Aim (0-2):

2: The aim and hypothesis were described;
1: Only the aim or hypothesis were described;
0: No aim and no hypothesis were provided.

Sample Selection (0-8)

Representativeness (0-2)

2: All individuals or random sampling: the sample represents the mean of the reference population (e.g., the sample, selected using systematic sampling methods, ensures adequate statistical power, even when referring to a single institution or community according to the study's purpose);
1: Non-random sampling: the sample is to some extent representative of the mean of the reference population (non-probability sampling methods);
0: A selected group of users, or no description of the sampling strategy.

Sample Size (0-2)

2: Justified and satisfactory (the method for defining the sample size is described and the size is appropriate);
1: Justified and not satisfactory, or not justified and satisfactory (e.g., the method for defining the sample size is described, but the size is not appropriate, or there is no method description, but the size is satisfactory);
0: Not justified (e.g., none of the above conditions is met).

Non-Response Rate (0-2)

2: Concurrent presence of 3 conditions: i) assessment of response rate, ii) satisfactory response rate (>70%), and iii) evaluation and comparability of respondents' and non-respondents' characteristics;
1: Under 2 out of 3 previous conditions (e.g., the response rate assessed was satisfactory, but the characteristics of nonrespondents were not evaluated);
0: Under 1 or no condition out of 3.

Exposure Assessment (0-2)

2: Validated measurement tool (e.g., validated questionnaires or direct measurements);
1: No validated measurement tool, but availability or description of the tool (e.g., a detailed description of items of a new non-validated questionnaire);
0: No availability or description of the measurement tool.

Comparability (0-2)

Control of Confounding Factors (0-1)

1: In the study, factors that may influence other variables, distorting the results, are controlled (e.g., use of ANCOVAs or statistically adjusted variables for sex, age, education);
0: No control.

Comparability of Groups (0-1)

1: The study individuals in the different groups are comparable (e.g., sex, age, education composition or differences were controlled);
0: The study individuals in the different groups are not comparable.

Outcome (0-4)

Assessment of the Outcome (0-2)

2: Independent or double-blind study
1: Self-report, single-blind study;
0: No description.

Statistics (0-2)

2: Concurrent presence of 3 conditions: i) statistical tests for data analysis are clearly described; ii) statistical tests are appropriate; and iii) presence of a measure of association (confidence intervals or p-value);
1: Under 2 out of 3 previous conditions;
0: Under 1 or no condition out of 3.

Total Score ___/16

13-16 stars: high quality and low risk of bias; 9-12 stars: moderate quality and moderate risk of bias; 5-8 stars: low quality and high risk of bias; 4 or fewer stars: unsatisfactory.
